# On Contusions of Muscles

**Published:** 1843-04

**Authors:** Wm. Allison


*On Contusions of Muscles.*—By Wm. Allison.—The most interesting circumstance connected with contusions of muscles is the difficulty of distinguishing those injuries from dislocations or fractures of those bones which form cups for joints. Muscles are bruised by falls or blows; a limb is consequently stiff (whilst lenghtened or shortened), and it becomes motionless at a joint, so that neither flexion nor extension can be performed by the subject of the accident; and sometimes considerable swelling ensues before a medical man arrives. The surgeon's attempt to move the limb, in order to ascertain the nature of the injury, produces a painful spasmodic action of muscles, sometimes without proving advantageous to himself, in his endeavour to find out the precise cause of the loss of muscular action and the stiffness of the limb. And whilst the surgeon has no means of completely satisfying himself with respect to the accident, he is closely questioned by the patient and his friends, and must either express his doubts or give indirect answers. We all know that by sleeping with the head upon the arm, so as to make good pressure on the median nerve, we may become unable, during many minutes after waking, to move the fore-arm; that by sleeping cross-legged in a chair, so as to make a firm pressure upon the popliteal nerve, we may be, during many minutes, unable to stand upon the leg; that rheumatic stiffness may require great muscular efforts to restore the use of the limbs; and that sometimes after fractures of the arm or thigh, one means only can overcome the muscular rigidity, and restore action—namely, the frequent, resolute efforts of the patient to put the muscles in action; but I think it behoves us especially to ascertain the different effects and the practical consequences of violent muscular contusions.

1. The muscle or muscles may be so bruised as to be simply benumbed (with tonic or permanent contraction or with relaxation), the nerves being affected by the fall or blow, something like the brain from concussion.

Case I.—Mr. Smith, of this town, remained with his leg, for half an hour, under a horse which had fallen with him, and which had then laid upon him, the horse having made fruitless attempts to get up whilst the leg was under him. Mr. Smith could not move his leg when first lifted up; but, being supported, he made great efforts to use it, until in ten or fifteen minutes he gradually became able to walk.

Case II.—A woman, named Parkin, of Ordsall, fell from a load of hay upon the hard ground, in a very hot, dry summer; her thigh was for some weeks in the exact position of a dislocation into the ischiatic notch. By forcible extension I could place the limb in the natural position without pain; but it always returned to the apparently dislocated position. No fracture of the acetabulum nor of the neck of the thigh-bone could be felt. In four or five weeks she recovered the use of the limb.

2. The muscles may be bruised whilst in action, and remain stiff (with atonic contraction or with relaxation) so long as they are left at rest; but the moment an attempt is made by the patient or surgeon to move the limb, a violent, painful quivering or irregular spasmodic action comes on, and the limb cannot be placed in the natural position.

Case III.—A boy was carrying two pails full of water suspended from his shoulders; in attempting to step down with them, from a very highly-raised causeway, he slipped backwards and sideways upon the edge of the causeway, shooting his heels before him. On my arrival his leg presented the appearance of a dislocation upon the pubis. Every attempt to bring that knee to a level with the other, either on a mattrass or when standing upon the sound limb, failed; but it produced painful, spasmodic muscular action. The chief pain was in the groin, where there was a swelling; but as the head of the thigh-bone could not be felt there, I proclaimed the accident to be "a serious injury of the muscles," which probably would continue some weeks. By leeches, fomentations, &c., the boy recovered in a week.

3. Muscles may be bruised, with extravasation or some injury ending in suppuration.

Case IV.—I was called (July 1) to a lady who had been thrown out of a pony carriage in this town; her shoulder was dislocated, and her leg was bruised. Both before and after the dislocation was reduced she walked twenty or thirty yards very well, and she was sent home, a few miles off, in a chaise. The leg swelled, and became stiff and useless (to herself immovable). After leeches, fomentations, poultices, &c., had been used, with entire rest for upwards of five weeks, she became alarmingly ill, with high constitutional disturbance during her seventh month of pregnancy; and in about a week from that time (on the 17th of August) I opened a deep-seated abscess under the fascia of the gastrocnemius muscle, after which she became perfectly well, before her confinement (on the 17th of October), from which she recovered as usual.

4. Muscles may be bruised, with laceration of fibres.

Case V.—In June, 1839, I was desired to visit a stout, heavy, muscular man, who, it was supposed, had dislocated his hip. On my arrival I heard that, whilst sitting upon the shelvings of a cart, he fell backwards with his shoulders upon the wheel, and reached the ground (hard sand-rock) in about the sitting position. Moving the limb gave excruciating pain, and occasioned spasmodic muscular contraction; nevertheless, after having placed his shoulders and hips in a straight line upon a mattrass, and having grasped each ancle with one hand, I drew him downwards towards the bottom of the mattrass, when I found the inner ancle-bone of the injured side full an inch and a quarter below the other, with the heel inclining inwards. I could bend the knee upwards towards the abdomen, but could not cross one thigh over the other. Adduction could be effected with some difficulty; but this limb was always longer than the other by an inch and a quarter, with the knee separated, and the toes turned outwards when in the easiest position, and there was a constant pain in the perineum. If the case had been one of dislocation into the foramen ovale, I supposed adduction could not have been effected, and I was not aware that it could be any other variety of dislocation. There was no crepitus about the joint; I

therefore believed it to be lengthening of the limb, mentioned by the late Sir A. P. Cooper, and delivered my opinion decisively, "that there was not any dislocation." However, I felt much more satisfied after my partner had accompanied me on my next visit. To the question, "What *is* the accident?" we replied, "a rupture of some part of the muscle which forms the buttock."

The gentleman was bled in the arm, took an opiate, had his hip fomented, and warm, damp linen kept upon the painful part; he then took castor oil; on the following morning twelve leeches were applied, and afterwards poultices. We cannot lift patients so affected into and out of warm baths; he was kept in the easiest posture, &c., and the case went on quietly; but the lengthened state of the limb, the inability to move it without violent pain for some weeks, and the sensation of something in the perineum, gave rise to doubts amongst his friends respecting a dislocation. In this case extension of the rigid muscles after the second week, by pulleys applied as if for a dislocation into foramen ovale, until fainting was produced, appeared to be serviceable. The consequences of the accident were, not only that the limb gradually became of the same length as the other, but that contraction went on until it was about an inch shorter, as it remains to this day, that he halts in walking, and that he cannot ride on horse-back without making the hip and thigh muscles very painful. I have on several occasions seen limbs as rigid from falls and bruises, when all attempts at motion have given violent pain; but in this case I cannot account for the lengthening and subsequent shortening of the limb, but by a laceration of muscular fibres. He can now walk ten or fifteen miles in a day without fatigue.

In relating the foregoing cases, I may not have classed them correctly. For instance, the pregnant lady may have had some laceration of the deep-seated tissues of her leg, as the carriage wheel had evidently passed over it; but that being now doubtful, merely serves to show the difficulty of stating the precise extent of injury at the first visit after an accident.

A surgeon, called to reduce a dislocation, has to distinguish one from a fracture near the joint; and sometimes, in forming his diagnosis, he is perplexed by muscular rigidity; at other times by considerable tumefaction from extravasation of blood; and on some occasions by extreme tension from effusion, the consequence of inflammation. As the late Sir A. P. Cooper, when speaking of dislocation, said, "Few accidents are more likely to endanger the reputation of the surgeon, as the patient may become a living memorial of his ignorance." I shall not apologize either for having called the attention of surgeons in the commencement of their career to this particular part of their practice; or for reminding them further that the biceps tendon may be ruptured, or that it may be displaced from its natural situation in passing over the head of the os humeri.—*Amer. Journ.*, from *Prov. Med Journ.*, May 28, 1842.

